# The Mechanistic Classification of Addictive Drugs

**DOI:** 10.1371/journal.pmed.0030437

**Published:** 2006-11-14

**Authors:** Christian Lüscher, Mark A Ungless

## Abstract

The authors review recent research on the molecular mechanisms of addiction and propose a new classification for addictive drugs.

The consumption of a variety of natural and synthetic substances can lead to addiction, which is commonly defined by the loss of control and compulsive consumption despite negative consequences. Although addictive drugs have diverse molecular targets in the brain, they share the common initial effect of increasing the concentration of dopamine released from mesocorticolimbic projections.

In this article, we review recent research that has advanced our understanding of the molecular mechanisms underlying this increase of dopamine. Based on this research, we propose a new classification for addictive drugs that we believe may help in directing research towards more effective treatment of addiction (see Table 1 and Figure 1).

**Table 1:**
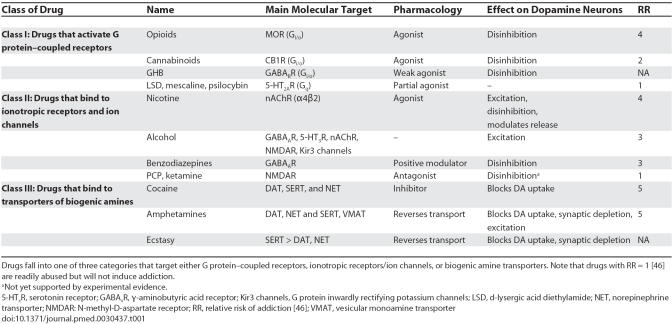
The Mechanistic Classification of Drugs of Abuse

**Figure 1:**
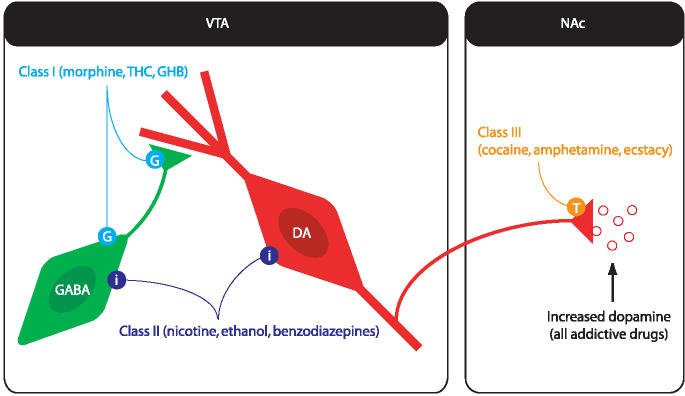
The Dominant Targets Involved in Increasing Dopamine for the Major Types of Addictive Drugs G, G_i/o_-coupled receptors; i, ionotropic receptors/ion channels; T, monoamine transporters

## Induction of Addiction

The mesocorticolimbic dopamine system originates in the ventral tegmental area (VTA), which projects most notably to the nucleus accumbens (NAc) and the prefrontal cortex (PFC). It is a defining commonality of all addictive drugs that they increase dopamine concentrations in target structures of the mesocorticolimbic projections [1,2]. The release of dopamine from these projections is thought to play a crucial role in the induction of compulsive addictive behaviour. The precise role of dopamine in reinforcement and the modulation of reward-related behaviour remains controversial [3]. Most experts in the field agree that some aspects of reward (e.g., euphoria/ pleasure) are dopamine-independent [4]. In rats, for example, blockade of mesolimbic DA (dopamine) signalling with either systemic or intra-NAc neuroleptic pre-treatment potentiated the sensitivity to nicotine's rewarding properties [5]. Also, dopamine-deficient mice display conditioned place preference for morphine [6].

Moreover, it is important to realize that, once compulsive use has been established, addiction is thought to be largely dopamine-independent. Nonetheless, it is widely accepted that the induction of addiction crucially involves mesocorticolimbic dopamine.

**Some of the Key Papers on the Cellular Effects of Addictive Drugs**

**Johnson and North, 1992** [11]: A classic paper demonstrating the disinhibitory effect of opioids on dopamine neurons.

**Cruz et al., 2004** [17]: A current model explaining how the popular club drug GHB activates VTA neurons via its action on the GABA_B_ receptor.

**Maskos et al., 2005** [21]: An elegant study showing that in knockout mice lacking the β2 subunit of the acetylcholine receptor, the rewarding properties of nicotine can be restored by selective re-expression in VTA neurons.

**Chen et al., 2006** [39]: A recent paper demonstrating that the rewarding properties of cocaine are absent in mice that express a cocaine-insensitive dopamine transporter.

**Ungless et al., 2001** [56]: The first in a series of papers to observe a form of long-term synaptic plasticity of glutamatergic synapses in the VTA in response to addictive drugs. This and other adaptive changes common to several addictive drugs downstream of the dopamine increase are the focus of much current research.

**Saal et al., 2003** [57]: In this paper the authors observe a form of long-term synaptic plasticity of glutamatergic synapses in the VTA in response to several addictive drugs. This and other adaptive changes downstream of the dopamine increase are the focus of much current research.

Taken together, these findings suggest that it may be possible to dissociate the hedonic value of a drug from its addictive properties using modern molecular tools. Such experiments, which may have important clinical ramifications, obviously depend on further mechanistic insight regarding drug action. We believe that our classification, based on the molecular and cellular mechanisms through which addictive drugs increase mesocorticolimbic dopamine, will provide the conceptual framework required to facilitate research to resolve these and related issues.

## The Classification

Addictive drugs are a chemically heterogeneous group with very distinct molecular targets. Moreover, an individual drug may have more than one molecular target. Here we will focus on those mechanisms that are directly responsible for the increase in dopamine concentration. We distinguish three groups of addictive drugs: (1) drugs that bind to G protein–coupled receptors (GPCRs)—this group includes the opioids, cannabinoids, and γ -hydroxy butyrate (GHB); (2) drugs that interact with ionotropic receptors or ion channels—this group includes nicotine, alcohol, and benzodiazepines; and (3) drugs that target monoamine transporters—this group comprises cocaine, amphetamine, and methylenedioxymetamphetamine (MDMA, ecstasy) (see Table 1 and Figure 1).

GPCRs that are of the G_i/o_ family inhibit neurons through post-synaptic hyperpolarisation and pre-synaptic regulation of the transmitter release. In the VTA, the action of these drugs is preferentially on the γ -aminobutyric acid (GABA) neurons that act as local inhibitory interneurons. They also inhibit glutamate release [7], but in the VTA their dominant mechanism of action is inhibition of GABA neurons leading to a net disinhibition of dopamine neurons and increased dopamine release. Addictive drugs that bind to ionotropic receptors and ion channels can have combined effects on dopamine neurons and GABA neurons, eventually leading to enhanced release of dopamine. Finally, addictive drugs interfering with monoamine transporters block the re-uptake of dopamine, or stimulate non-vesicular release of dopamine, causing an accumulation of extracellular dopamine in target structures. We will now discuss examples for each type of mechanism in detail.

## Class I: Drugs That Activate G_i/o-_Coupled Receptors

### Morphine and other opioids.

These strongly increase the release of mesolimbic dopamine by their action on μ-opioid receptors (MORs), which are expressed on inhibitory GABAergic interneurons of the VTA [8]. MORs have a dual action: they hyperpolarise GABA neurons and decrease GABA release. The post-synaptic hyperpolarisation is mediated by Kir3/ G protein–coupled inwardly rectifying K^+^ (GIRK) channels coupled to MORs on the soma and the dendrites, in analogy to other parts of the brain [9], while MORs expressed on the pre-synaptic terminals decrease release by inhibiting Ca^2+^ channels or activating voltage-gated K^+^ channels [10]. MORs in the two cellular compartments therefore rely on distinct effectors, which together lead to strong inhibition of GABA neurons and disinhibition of dopamine neurons [11].

### Delta-9-tetrahydrocannabinol.

Delta-9-tetrahydrocannabinol (THC) binds to type 1 cannabinoid receptors (CB1Rs) in the brain. In the VTA, these receptors are expressed on GABA neurons and on terminals of glutamatergic synapses on dopamine neurons [12]. Pharmacological application of THC causes a net disinhibition by decreasing the release of the neurotransmitter GABA in acute midbrain slices [13]. To date, no evidence is available to suggest that CB1Rs also activate Kir3/GIRK channels in these neurons.

### GHB.

This is an increasingly popular club drug that is readily self-administered and induces conditioned place preference (see Glossary) in animal models, and leads to addiction in humans [14]. GHB has two binding sites in the brain, but its pharmacological effects are absent in knockout mice lacking functional GABA_B_ receptors [15,16], suggesting that they are entirely mediated by these receptors. Although GABA_B_ receptors are expressed on both GABA and dopamine neurons of the VTA, GHB affects almost exclusively GABA neurons at concentrations typically obtained with recreational use. This happens because the coupling efficiency of Kir3/GIRK channels in dopamine neurons is very low (the EC_50_ differs by an order of magnitude between GABA and dopamine neurons), which in turn is due to the cell type–specific subunit expression of Kir3/GIRK channels [17]. Dopamine neurons lack GIRK_1_, but express GIRK_2_ and GIRK_3_, which when co-assembled have a lower affinity for the βγ-dimer of the G_i/o_ protein compared to channels that contain GIRK_1_ As a consequence, only GABA neurons are hyperpolarised at concentrations below 1 mM, causing a disinhibition of dopamine neurons.

## Class II: Drugs That Mediate Their Effects Via Ionotropic Receptors

### Nicotine.

This drug targets nicotinic acetylcholine receptors (nAChRs) in the brain. When nicotine binds nAChRs they become cation-permeable and depolarise the cell. Nicotine increases dopamine through a complex interplay of actions at these ionotropic receptors on GABA and dopamine neurons, and glutamatergic inputs to dopamine neurons [18]. Brief applications of nicotine to these neurons in rat brain slices causes a depolarisation and increased firing, although prolonged exposure leads to rapid receptor desensitisation [19]. In addition, following desensitisation of β2-containing nAChRs on GABA neurons, GABA release is decreased (i.e., the excitatory effect of endogenous acetylcholine is reduced), leading to a more prolonged disinhibition of dopamine neurons [20]. It is evident that β2-containing nAChRs are responsible for the rewarding effects of nicotine because β2 knockout mice do not self-administer nicotine and do not show nicotine-evoked dopamine release [21]. These deficits can be restored through in vivo transfection of the β2 subunit in the VTA [22].

This view is further complicated by two more actions of nicotine. Homomeric a7-containing nAChRs, which are mainly expressed on synaptic terminals of excitatory glutamatergic afferents onto dopamine neurons in the VTA, facilitate glutamate release [20]. This effect may also contribute to nicotine-evoked dopamine release and/or the long-term changes induced by the drugs related to addiction (e.g., long-term synaptic potentiation of excitatory inputs). Furthermore, recent evidence suggests that nicotine directly modulates dopamine release in the NAc [23,24].

### Benzodiazepines.

Benzodiazepines (BZD) increase mesocorticolimbic dopamine and can lead to addiction. BZD are positive modulators of the GABA_A_ receptor. When injected into the VTA, the GABA_A_ receptor agonist muscimol seems to inhibit interneurons more efficiently compared to dopamine neurons, which may lead to a net disinhibition of the dopamine neurons [25]. This selectivity may relate to cell-type specific subunit expression. For example, when dopamine neurons were isolated from the VTA of transgenic mice that express green fluorescent protein under the control of the tyrosine hydroxylase gene promoter, reverse transcriptase–PCR analysis revealed the presence of a2, a3, and a4 subunits. Conversely, a1 was the major subunit expressed in GABA neurons [26].

### Ethanol.

This drug has a complex pharmacology. No single receptor mediates all of the effects of alcohol [27]. On the contrary, alcohol alters the function of a number of receptors and cellular functions, including GABA_A_ receptors [28], Kir3/GIRK [29,30] and other K channels [31], I_h_ [32], N-methyl-D-aspartate (NMDA) receptors [33], nAChRs [34], and 5-HT_3_ receptors [35]. In addition, ethanol also interferes with adenosine re-uptake by inhibiting the equilibrative nucleoside transporter ENT1, although it is not clear if this plays a role in ethanol-induced dopamine release [36]. How ethanol causes the increase in dopamine remains unclear. Possibilities include a net disinhibition similar to that proposed for benzodiazepines or direct depolarisation, for example by inhibition of a K channel [31].

## Class III: Drugs That Bind to Transporters of Biogenic Amines

### Cocaine.

In the central nervous system, cocaine blocks dopamine, noradrenaline, and serotonin uptake through inhibition of their respective transporters. Blocking of the dopamine transporter (DAT) leads to an increase of dopamine concentrations in the nucleus accumbens. (The firing rate of DA neurons of the VTA actually decreases with cocaine application, which is due to the effects of dopamine on D2 autoreceptors on DA neurons [37].) In mice lacking DAT, dopamine still increases in response to cocaine [38], which could be the result of inhibition of dopamine uptake by other monoamine transporters. Consistent with this suggestion, DAT knockout mice still self-administer cocaine, and this behaviour is abolished in combined DAT– serotonin transporter (SERT) knockout mice [39]. SERT-mediated re-uptake of dopamine only occurs in situations where dopamine levels are already high, as in DAT knockout mice. This is confirmed by a study that used a knock-in mouse line carrying a functional DAT that was insensitive to cocaine. In these mice, cocaine did not elevate extracellular dopamine in the nucleus accumbens, and did not produce reward, as measured by conditioned place preference [40]. Finally, it is important to point out that selective SERT inhibition in humans (e.g., fluoxetine to treat depression) does not carry any addiction liability.

### Amphetamine, methamphetamine, and their many derivates.

These exert their effects by reversing the action of biogenic amine transporters at the plasma membrane [41]. Amphetamines are substrates of these transporters and are taken up into the cell. Every molecule that is taken up generates a current causing a depolarisation of the dopamine neurons, which could contribute to enhanced dopamine release [42]. In addition, once in the cell, amphetamines interfere with the vesicular monoamine transporter, depleting synaptic vesicles. As a consequence, dopamine increases in the cytoplasm from where it is released by plasma membrane transporters working in reverse. In other words, normal vesicular release of dopamine decreases (i.e., synaptic vesicles contains less transmitter, the quantal content becomes smaller), while non-vesicular release increases. Similar mechanisms apply for other biogenic amines such as serotonin and norepinephrine.

### Methylenedioxymetamphetamine (ecstasy).

As for the amphetamines, MDMA causes the release of biogenic amines by reversing the action of their respective transporters. Although MDMA has a preferential affinity for SERTs and therefore increases the extracellular concentration of serotonin, it also strongly increases dopamine [43].

## Drugs of Abuse Yet to Be Classified

There are a number of abused drugs about which there is no clear consensus concerning their addictive properties (e.g., hallucinogens and dissociative anaesthetics). For example, LSD, which is widely abused, does not appear to be addictive. Animals will not self-administer hallucinogens, suggesting that they are not rewarding [44]. Importantly, these drugs fail to evoke dopamine release, further supporting the idea that only drugs that activate the mesolimbic dopamine system are addictive. Instead, the critical action of hallucinogens may be increased glutamate release in the cortex, presumably through a pre-synaptic effect on 5-HT_2A_ receptors expressed on excitatory afferents from the thalamus [45].

**Glossary**

**Conditioned place preference:** A behavioural test for examining the rewarding properties of drugs. The preference of a particular environment associated with drug exposure is measured by comparing the time an animal spends in the compartment where the drug was previously administered compared to a control compartment.

**Coupling efficiency:** The efficiency with which a given G protein–coupled receptor can activate an effector.

**DARP32:** Dopamine and cAMP-regulated phosphoprotein. A key target protein for increased dopamine that plays a role in signalling the effects of many addictive drugs.

**DeltaFosB:** A transcription factor that is induced in areas such as the NAc in response to many addictive drugs, and thought to be involved in the long-term maintenance of addictive behaviour.

**EC_50_**: 50% effective concentration, i.e., the concentration of an agonist that produces 50% of the maximal effect.

**Equilibrative nucleoside transporter ENT1:** Transporter responsible for the re-uptake of adenosine.

**Homomeric α7-containing nAChRs:** Nicotinic acetylcholine receptors formed by five subunits of the α7 type.

**Kir3/GIRK channels:** One class of inwardly rectifying potassium channels; Kir3 are also termed G protein–coupled inwardly rectifying K^+^ channels.

**Quantal content:** The amount of neurotransmitter released by a single vesicle.

The main effect of the NMDA receptor antagonists phencyclidine (PCP) and ketamine are feelings of separation of mind and body and, at higher doses, stupor and coma, which is why they are called dissociative anaesthetics. Based on early assessments, NMDA receptor antagonists have been classified as non-addictive drugs of abuse [46]. This classification has recently been questioned for PCP. For example, PCP has some reinforcing properties in rodents when applied directly to the NAc and the PFC [47]. Moreover, increased dopamine levels were measured in vivo with micro-dialysis after systemic or PFC injection of PCP in freely moving rats. Similar results were also obtained with local injections of MK-801, a more selective and potent NMDA receptor antagonist than PCP, which supports the conclusion that PCP's effect on dopamine is mediated via the inhibition of NMDA receptors [48]. In this case, PCP would be a Class II drug according to our classification.

Inhalant abuse is defined by the recreational exposure to chemical vapours, such as nitrates, ketones, and aliphatic and aromatic hydrocarbons. In some countries it is particularly common among children, and some chemicals do induce addiction [49]. The mechanism of action remains unknown for most volatile substances. A very limited literature provides evidence that some inhalants alter the function of ionotropic receptors and ion channels throughout the central nervous system [50]. Nitrous oxide, for example, binds to NMDA receptors [51,52] and fuel additives enhance the GABA_A_ receptor function [53]. Toluene increases firing in VTA neurons [54] and causes conditioned place preference [55]. Others, such as amyl nitrite (“poppers”), primarily produce smooth muscle dilatation, and enhance erection, but are not addictive. While this literature suggests that some inhalants may be Class II addictive drugs, clearly more research will be needed to confirm this choice.

## Implications for Research

We have presented a new mechanistic classification system for addictive drugs. There are a number of key features of this system. First, there are three types of mechanism. Second, each addictive drug only activates the dopamine system through a single mechanism (with the possible exception of ethanol, which has multiple molecular targets whose relative contributions to addiction remain elusive). Third, within each type of mechanism the effect on the dopamine system is similar (e.g., Class I drugs all activate dopamine neurons via disinhibition).

Although substantial progress into unravelling the neurobiological bases of addiction has been made, many open questions remain and few effective treatments are currently available. Much current research is therefore aimed at understanding the neuroadaptive changes induced by addictive drugs, such as increased expression of deltaFosB and DARP32 [1] or the effects on excitatory glutamate transmission [56–58]. The present classification represents a framework that will facilitate research aiming at understanding how each drug induces the adaptive changes listed above and predicts that drugs of the same group are likely to share similar mechanisms.

## Implications for Developing Better Treatments for Addiction

Understanding the early phases of the induction of adaptive processes will also be important for the discovery of novel pharmacological treatment strategies. If activation of the dopamine system is indeed crucial for the development of addiction, then an interesting strategy may be to inhibit the mesocorticolimbic DA system (either pharmacologically or through direct stimulation). This idea is further supported by the observation that increases in dopamine play an important role in relapse, particularly drug-induced relapse [59,60]. In this context, the present classification would also serve to identify and organise treatments at the level of the VTA. For example, naloxone will block the effect of opioids, while the high affinity GABA_B_ receptor agonist baclofen would inhibit GABA and dopamine neurons, thus efficiently blocking DA release.

Treatments in use, or at pre-clinical stages of development, are either drug-specific (e.g., vaccines or antagonists that directly block drug action, or agonists for use as drug substitutes) or target a mechanism that is common to several drugs (e.g., medications that reduce craving in multiple forms of addiction) [57]. Many new addiction treatments (for a comprehensive list of approved and experimental medications see [61]) appear to operate downstream of initial targets (e.g., naltrexone or acamprosate for opiate and alcohol addiction), although their precise mechanisms of action are not entirely clear. Our classification points to a third approach of developing treatments for different classes of drugs based on the mechanisms through which they increase dopamine. For example, targeting the DAT should be useful in treating addiction to any Class III drug. The same may be true for future treatments that interfere with G protein–coupled signalling—such treatments may be useful for all Class I drugs.

Finally, we hope that our strikingly simple mechanistic classification will provide students and clinicians with a useful conceptual framework for understanding a diverse and often complex literature concerning such an important medical issue.

## Abbreviations

CB1R: type 1 cannabinoid receptor
DA: dopamine
DAT: dopamine transporter
GABA: γ -aminobutyric acid
GHB: γ -hydroxy butyrate
GIRK: G protein–coupled inwardly rectifying K^+^
GPCR: G protein–coupled receptor
MDMA: methylenedioxymetamphetamine
MOR: μ-opioid receptor
NAc: nucleus accumbens
nAChR: nicotinic acetylcholine receptor
NMDA: N-methyl-D-aspartate
PCP: phencyclidine
PFC: prefrontal cortex
SERT: serotonin transporter
THC: delta-9-tetrahydrocannabinol
VTA: ventral tegmental area

## References

[pmed-0030437-b001] Nestler EJ (2005). Is there a common molecular pathway for addiction?. Nat Neurosci.

[pmed-0030437-b002] Pierce RC, Kumaresan V (2006). The mesolimbic dopamine system: The final common pathway for the reinforcing effect of drugs of abuse?. Neurosci Biobehav Rev.

[pmed-0030437-b003] Berridge KC, Robinson TE (1998). What is the role of dopamine in reward: Hedonic impact, reward learning, or incentive salience?. Brain Res Brain Res Rev.

[pmed-0030437-b004] Bechara A, Nader K, van der Kooy D (1998). A two-separate-motivational-systems hypothesis of opioid addiction. Pharmacol Biochem Behav.

[pmed-0030437-b005] Laviolette SR, van der Kooy D (2003). Blockade of mesolimbic dopamine transmission dramatically increases sensitivity to the rewarding effects of nicotine in the ventral tegmental area. Mol Psychiatry.

[pmed-0030437-b006] Hnasko TS, Sotak BN, Palmiter RD (2005). Morphine reward in dopamine-deficient mice. Nature.

[pmed-0030437-b007] Meir A, Ginsburg S, Butkevich A, Kachalsky SG, Kaiserman I (1999). Ion channels in presynaptic nerve terminals and control of transmitter release. Physiol Rev.

[pmed-0030437-b008] Pickel VM, Garzon M, Mengual E (2002). Electron microscopic immunolabeling of transporters and receptors identifies transmitter-specific functional sites envisioned in Cajal's neuron. Prog Brain Res.

[pmed-0030437-b009] Lüscher C, Jan LY, Stoffel M, Malenka RC, Nicoll RA (1997). G protein-coupled inwardly rectifying K^+^ channels (GIRKs) mediate postsynaptic but not presynaptic transmitter actions in hippocampal neurons. Neuron.

[pmed-0030437-b010] Vaughan CW, Ingram SL, Connor MA, Christie MJ (1997). How opioids inhibit GABA-mediated neurotransmission. Nature.

[pmed-0030437-b011] Johnson SW, North RA (1992). Opioids excite dopamine neurons by hyperpolarization of local interneurons. J Neurosci.

[pmed-0030437-b012] Melis M, Pistis M, Perra S, Muntoni AL, Pillolla G (2004). Endocannabinoids mediate presynaptic inhibition of glutamatergic transmission in rat ventral tegmental area dopamine neurons through activation of CB1 receptors. J Neurosci.

[pmed-0030437-b013] Szabo B, Siemes S, Wallmichrath I (2002). Inhibition of GABAergic neurotransmission in the ventral tegmental area by cannabinoids. Eur J Neurosci.

[pmed-0030437-b014] Snead OC, Gibson KM (2005). Gamma-hydroxybutyric acid. N Engl J Med.

[pmed-0030437-b015] Quéva C, Bremmer-Danielsen M, Edlund A, Ekstrand AJ, Elg S (2003). Effects of GABA agonists on body temperature regulation in GABA(B(1))-/- mice. Br J Pharmacol.

[pmed-0030437-b016] Kaupmann K, Cryan JF, Wellendroph P, Mombereau C, Sansig G (2003). Specific gamma-hydroxybutyrate-binding sites but loss of pharmacological effects of gamma-hydroxybutyrate in GABA(B)(1)-deficient mice. Eur J Neurosci.

[pmed-0030437-b017] Cruz HG, Ivanova T, Lunn ML, Stoffel M, Slesinger PA (2004). Bi-directional effects of GABA(B) receptor agonists on the mesolimbic dopamine system. Nat Neurosci.

[pmed-0030437-b018] Fagen ZM, Mansvelder HD, Keath JR, McGehee DS (2003). Short- and long-term modulation of synaptic inputs to brain reward areas by nicotine. Ann N Y Acad Sci.

[pmed-0030437-b019] Pidoplichko VI, DeBiasi M, Williams JT, Dani JA (1997). Nicotine activates and desensitizes midbrain dopamine neurons. Nature.

[pmed-0030437-b020] Mansvelder HD, Keath JR, McGehee DS (2002). Synaptic mechanisms underlie nicotine-induced excitability of brain reward areas. Neuron.

[pmed-0030437-b021] Picciotto MR, Zoli M, Rimondini R, Lena C, Marubio LM (1998). Acetylcholine receptors containing the beta2 subunit are involved in the reinforcing properties of nicotine. Nature.

[pmed-0030437-b022] Maskos U, Molles BE, Pons S, Besson M, Guiard BP (2005). Nicotine reinforcement and cognition restored by targeted expression of nicotinic receptors. Nature.

[pmed-0030437-b023] Rice ME, Cragg SJ (2004). Nicotine amplifies reward-related dopamine signals in striatum. Nat Neurosci.

[pmed-0030437-b024] Zhang H, Sulzer D (2004). Frequency-dependent modulation of dopamine release by nicotine. Nat Neurosci.

[pmed-0030437-b025] Kalivas PW, Duffy P, Eberhardt H (1990). Modulation of A10 dopamine neurons by gamma-aminobutyric acid agonists. J Pharmacol Exp Ther.

[pmed-0030437-b026] Okada H, Matsushita N, Kobayashi K, Kobayashi K (2004). Identification of GABAA receptor subunit variants in midbrain dopaminergic neurons. J Neurochem.

[pmed-0030437-b027] Koob GF, Roberts AJ, Schulteis G, Parsons LH, Heyser CJ (1998). Neurocircuitry targets in ethanol reward and dependence. Alcohol Clin Exp Res.

[pmed-0030437-b028] Morrow AL (1995). Regulation of GABAA receptor function and gene expression in the central nervous system. Int Rev Neurobiol.

[pmed-0030437-b029] Kobayashi T, Ikeda K, Kojima H, Niki H, Yano R (1999). Ethanol opens G-protein-activated inwardly rectifying K+ channels. Nat Neurosci.

[pmed-0030437-b030] Lewohl JM, Wilson WR, Mayfield RD, Brozowski SJ, Morrisett RA (1999). G-protein-coupled inwardly rectifying potassium channels are targets of alcohol action. Nat Neurosci.

[pmed-0030437-b031] Appel SB, Liu Z, McElvain MA, Brodie MS (2003). Ethanol excitation of dopaminergic ventral tegmental area neurons is blocked by quinidine. J Pharmacol Exp Ther.

[pmed-0030437-b032] Okamoto T, Harnett MT, Morikawa H (2005). Hyperpolarization-activated cation current (Ih) is an ethanol target in midbrain dopamine neurons of mice. J Neurophysiol.

[pmed-0030437-b033] Krystal JH, Petrakis IL, Mason G, Trevisan L, D'Souza DC (2003). N-methyl-D-aspartate glutamate receptors and alcoholism: Reward, dependence, treatment, and vulnerability. Pharmacol Ther.

[pmed-0030437-b034] Soderpalm B, Ericson M, Olausson P, Blomqvist O, Engel JA (2000). Nicotinic mechanisms involved in the dopamine activating and reinforcing properties of ethanol. Behav Brain Res.

[pmed-0030437-b035] Lovinger DM (1999). 5-HT3 receptors and the neural actions of alcohols: An increasingly exciting topic. Neurochem Int.

[pmed-0030437-b036] Choi DS, Cascini MG, Mailliard W, Young H, Paredes P (2004). The type 1 equilibrative nucleoside transporter regulates ethanol intoxication and preference. Nat Neurosci.

[pmed-0030437-b037] Brodie MS, Dunwiddie TV (1990). Cocaine effects in the ventral tegmental area: Evidence for an indirect dopaminergic mechanism of action. Naunyn Schmiedebergs Arch Pharmacol.

[pmed-0030437-b038] Rocha BA, Fumagalli F, Gainetdinov RR, Jones SR, Ator R (1998). Cocaine self-administration in dopamine-transporter knockout mice. Nat Neurosci.

[pmed-0030437-b039] Rocha BA (2003). Stimulant and reinforcing effects of cocaine in monoamine transporter knockout mice. Eur J Pharmacol.

[pmed-0030437-b040] Chen R, Tilley MR, Wei H, Zhou F, Zhou FM (2006). Abolished cocaine reward in mice with a cocaine-insensitive dopamine transporter. Proc Natl Acad Sci U S A.

[pmed-0030437-b041] Sulzer D, Sonders MS, Poulsen NW, Galli A (2005). Mechanisms of neurotransmitter release by amphetamines: A review. Prog Neurobiol.

[pmed-0030437-b042] Ingram SL, Prasad BM, Amara SG (2002). Dopamine transporter-mediated conductances increase excitability of midbrain dopamine neurons. Nat Neurosci.

[pmed-0030437-b043] Morton J (2005). Ecstasy: Pharmacology and neurotoxicity. Curr Opin Pharmacol.

[pmed-0030437-b044] Nichols DE (2004). Hallucinogens. Pharmacol Ther.

[pmed-0030437-b045] Aghajanian GK, Marek GJ (1999). Serotonin and hallucinogens. Neuropsychopharmacology.

[pmed-0030437-b046] Goldstein A, Kalant H (1990). Drug policy: Striking the right balance. Science.

[pmed-0030437-b047] Carlezon WA, Wise RA (1996). Rewarding actions of phencyclidine and related drugs in nucleus accumbens shell and frontal cortex. J Neurosci.

[pmed-0030437-b048] Hondo H, Yonezawa Y, Nakahara T, Nakamura K, Hirano M (1994). Effect of phencyclidine on dopamine release in the rat prefrontal cortex; An in vivo microdialysis study. Brain Res.

[pmed-0030437-b049] Ridenour TA (2005). Inhalants: Not to be taken lightly anymore. Curr Opin Psychiatry.

[pmed-0030437-b050] Campagna JA, Miller KW, Forman SA (2003). Mechanisms of actions of inhaled anesthetics. N Engl J Med.

[pmed-0030437-b051] Mennerick S, Jevtovic-Todorovic V, Todorovic SM, Shen W, Olney JW (1998). Effect of nitrous oxide on excitatory and inhibitory synaptic transmission in hippocampal cultures. J Neurosci.

[pmed-0030437-b052] Nagele P, Metz LB, Crowder CM (2004). Nitrous oxide (N(2)O) requires the N-methyl-D-aspartate receptor for its action in Caenorhabditis elegans. Proc Natl Acad Sci U S A.

[pmed-0030437-b053] Martin JV, Iyer SV, McIlroy PJ, Iba MM (2004). Influence of oxygenated fuel additives and their metabolites on gamma-aminobutyric acidA (GABAA) receptor function in rat brain synaptoneurosomes. Toxicol Lett.

[pmed-0030437-b054] Riegel AC, French ED (2002). Abused inhalants and central reward pathways: Electrophysiological and behavioral studies in the rat. Ann N Y Acad Sci.

[pmed-0030437-b055] Gerasimov MR, Collier L, Ferrieri A, Alexoff D, Lee D (2003). Toluene inhalation produces a conditioned place preference in rats. Eur J Pharmacol.

[pmed-0030437-b056] Ungless MA, Whistler JL, Malenka RC, Bonci A (2001). Single cocaine exposure in vivo induces long-term potentiation in dopamine neurons. Nature.

[pmed-0030437-b057] Saal D, Dong Y, Bonci A, Malenka RC (2003). Drugs of abuse and stress trigger a common synaptic adaptation in dopamine neurons. Neuron.

[pmed-0030437-b058] Bellone C, Lüscher C (2006). Cocaine triggered AMPA receptor redistribution is reversed in vivo by mGluR-dependent long-term depression. Nat Neurosci.

[pmed-0030437-b059] Phillips PE, Stuber GD, Heien ML, Wightman RM, Carelli RM (2003). Subsecond dopamine release promotes cocaine seeking. Nature.

[pmed-0030437-b060] Kalivas PW, McFarland K (2003). Brain circuitry and the reinstatement of cocaine-seeking behavior. Psychopharmacology (Berl).

[pmed-0030437-b061] Volkow ND, Li TK (2004). Drug addiction: The neurobiology of behaviour gone awry. Nat Rev Neurosci.

